# Hardware-accelerated interactive data visualization for neuroscience in Python

**DOI:** 10.3389/fninf.2013.00036

**Published:** 2013-12-19

**Authors:** Cyrille Rossant, Kenneth D. Harris

**Affiliations:** Cortical Processing Laboratory, University College LondonLondon, UK

**Keywords:** data visualization, graphics card, OpenGL, Python, electrophysiology

## Abstract

Large datasets are becoming more and more common in science, particularly in neuroscience where experimental techniques are rapidly evolving. Obtaining interpretable results from raw data can sometimes be done automatically; however, there are numerous situations where there is a need, at all processing stages, to visualize the data in an interactive way. This enables the scientist to gain intuition, discover unexpected patterns, and find guidance about subsequent analysis steps. Existing visualization tools mostly focus on static publication-quality figures and do not support interactive visualization of large datasets. While working on Python software for visualization of neurophysiological data, we developed techniques to leverage the computational power of modern graphics cards for high-performance interactive data visualization. We were able to achieve very high performance despite the interpreted and dynamic nature of Python, by using state-of-the-art, fast libraries such as NumPy, PyOpenGL, and PyTables. We present applications of these methods to visualization of neurophysiological data. We believe our tools will be useful in a broad range of domains, in neuroscience and beyond, where there is an increasing need for scalable and fast interactive visualization.

## 1. Introduction

In many scientific fields, the amount of data generated by modern experiments is growing at an increasing pace. Notable data-driven neuroscientific areas and technologies include brain imaging (Basser et al., 1994; Huettel et al., 2004), scanning electron microscopy (Denk and Horstmann, 2004; Horstmann et al., 2012), next-generation DNA sequencing (Shendure and Ji, 2008), high-channel-count electrophysiology (Buzsáki, 2004), amongst others. This trend is confirmed by ongoing large-scale projects such as the Human Connectome Project (Van Essen et al., 2012), the Allen Human Brain Atlas (Shen et al., 2012), the Human Brain Project (Markram, 2012), the Brain Initiative (Insel et al., 2013), whose specific aims entail generating massive amounts of data. Getting the data, while technically highly challenging, is only the first step in the scientific process. For useful information to be inferred, effective data analysis and visualization is necessary.

It is often extremely useful to visualize raw data right after they have been obtained, as this allows scientists to make intuitive inferences about the data, or find unexpected patterns, etc. Yet, most existing visualization tools (such as matplotlib,^1^ Chaco,^2^ PyQwt,^3^ Bokeh,^4^ to name only a few Python libraries) are either focused on statistical quantities, or they do not scale well to very large datasets (i.e., containing more than one million points). With the increasing amount of scientific data comes a more and more pressing need for scalable and fast visualization tools.

The Python scientific ecosystem is highly popular in science (Oliphant, 2007), notably in neuroscience (Koetter et al., 2008), as it is a solid and open scientific computing and visualization framework. In particular, matplotlib is a rich, flexible and highly powerful software for scientific visualization (Hunter, 2007). However, it does not scale well to very large datasets. The same limitation applies to most existing visualization libraries.

One of the main reasons behind these limitations stems from the fact that these tools are traditionally written for central processing units (CPUs). All modern computers include a dedicated electronic circuit for graphics called a graphics processing unit (GPU) (Owens et al., 2008). GPUs are routinely used in video games and 3D modeling, but rarely in traditional scientific visualization applications (except in domains involving 3D models). Yet, not only are GPUs far more powerful than CPUs in terms of computational performance, but they are also specifically designed for real-time visualization applications.

In this paper, we describe how to use OpenGL (Woo et al., 1999), an open standard for hardware-accelerated interactive graphics, for scientific visualization in Python, and note the role of the programmable pipeline and shaders for this purpose. We also give some techniques which allow very high performance despite the interpreted nature of Python. Finally, we present an experimental open-source Python toolkit for interactive visualization, which we name Galry, and we give examples of its applications in visualizing neurophysiological data.

## 2. Materials and methods

In this section, we describe techniques for creating hardware-accelerated interactive data visualization applications in Python and OpenGL. We give a brief high-level overview of the OpenGL pipeline before describing how programmable shaders, originally designed for custom 3D rendering effects, can be highly advantageous for data visualization (Bailey, 2009). Finally, we apply these techniques to the visualization of neurophysiological data.

### 2.1. The OpenGL pipeline

A GPU contains a large number (hundreds to thousands) of execution units specialized in parallel arithmetic operations (Hong and Kim, 2009). This architecture is well adapted to realtime graphics processing. Very often, the same mathematical operation is applied on all vertices or pixels; for example, when the camera moves in a three-dimensional scene, the same transformation matrix is applied on all points. This massively parallel architecture explains the very high computational power of GPUs.

OpenGL is the industry standard for real-time hardware-accelerated graphics rendering, commonly used in video games and 3D modeling software (Woo et al., 1999). This open specification is supported on every major operating system^5^ and most devices from the three major GPU vendors (NVIDIA, AMD, Intel) (Jon Peddie Research, 2013). This is a strong advantage of OpenGL over other graphical APIs such as DirectX (a proprietary technology maintained by Microsoft), or general-purpose GPU programming frameworks such as CUDA (a proprietary technology maintained by NVIDIA Corporation). Scientists tend to favor open standard to proprietary solutions for reasons of vendor lock-in and concerns about the longevity of the technology.

**Figure 1:**
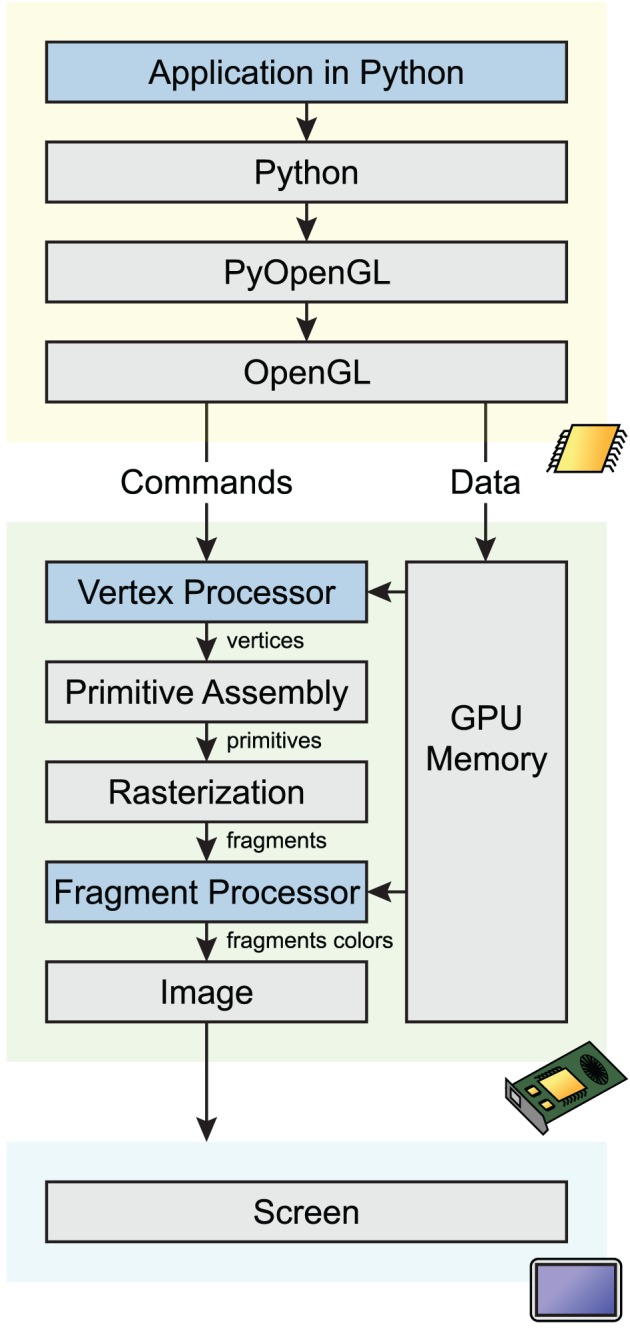
**Simplified OpenGL pipeline**. Graphical commands and data go through multiple stages from the application code in Python to the screen. The code calls OpenGL commands and sends data on the GPU through PyOpenGL, a Python-OpenGL binding library. Vertex shaders process data vertices in parallel, and return points in homogeneous coordinates. During rasterization, a bitmap image is created from the vector primitives. The fragment shader processes pixels in parallel, and assigns a color and depth to every drawn pixel. The image is finally rendered on screen.

OpenGL defines a complex pipeline that describes how 2D/3D data is processed in parallel on the GPU before the final image is rendered on screen. We give a simplified overview of this pipeline here (see Figure 1). In the first step, raw data (typically, points in the original data coordinate system) are transformed by the vertex processor into 3D vertices. Then, the primitive assembly creates points, lines and triangles from these data. During rasterization, these primitives are converted into pixels (also called fragments). Finally, those fragments are transformed by the fragment processor to form the final image.

An OpenGL Python wrapper called PyOpenGL allows the creation of OpenGL-based applications in Python (Fletcher and Liebscher, 2005). A critical issue is performance, as there is a slight overhead with any OpenGL API call, especially from Python. This problem can be solved by minimizing the number of OpenGL API calls using different techniques. First, multiple primitives of the same type can be displayed efficiently via batched rendering. Also, PyOpenGL allows the transfer of potentially large NumPy arrays (Van Der Walt et al., 2011) from host memory to GPU memory with minimal overhead. Another technique concerns shaders as discussed below.

### 2.2. OpenGL programmable shaders

Prior to OpenGL 2.0 (Segal and Akeley, 2004), released in 2004, vertex and fragment processing were implemented in the *fixed-function pipeline*. Data and image processing algorithms were described in terms of predefined stages implemented on non-programmable dedicated hardware on the GPU. This architecture resulted in limited customization and high complexity; as a result, a programmable pipeline was proposed in the core specification of OpenGL 2.0. This made it possible to implement entirely customized stages of the pipeline in a language close to C called the *OpenGL Shading Language* (GLSL) (Kessenich et al., 2004). These stages encompass most notably vertex processing, implemented in the *vertex shader*, and fragment processing, implemented in the *fragment shader*. Other types of shaders exist, like the geometry shader, but they are currently less widely supported on standard hardware. The fixed-function pipeline has been deprecated since OpenGL 3.0.

The main purpose of programmable shaders is to offer high flexibility in transformation, lighting, or post-processing effects in 3D real-time scenes. However, being fully programmable, shaders can also be used to implement arbitrary data transformations on the GPU in 2D or 3D scenes. In particular, shaders can be immensely useful for high-performance interactive 2D/3D data visualization.

**Figure 2:**
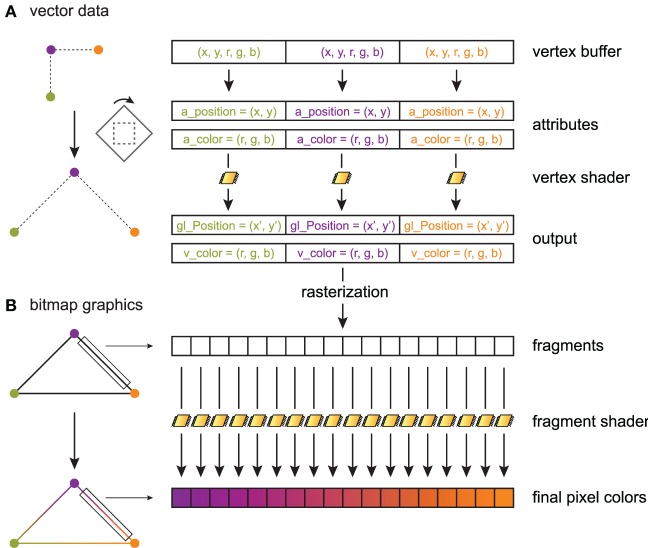
**Toy example illustrating shader processing**. Three connected line segments forming a triangle are rendered from three vertices. The triangle is linearly transformed, and color gradients are applied to the segments. **(A)** Three 5D data points are stored in a GPU vertex buffer object. Each point is composed of two scalar coordinates x and y (2D space), and three scalar RGB color components. The vertex shader processes these points in parallel on the graphics card. For each point, the vertex shader returns a point in homogeneous coordinates (here, two coordinates x′ and y′), along with *varying* variables (here, the point's color) that are passed to the fragment shader. The vertex shader implements linear or non-linear transformations (here, a rotation and a scaling) and is written in GLSL. Primitives (here, three line segments) are constructed in the primitive assembly. **(B)** During rasterization, a bitmap image is created out of these primitives. The fragment shader assigns a color to every drawn pixel in parallel. Varying variables passed by the vertex shader to the fragment shader are interpolated between vertices, which permits, for example, color gradients.

The principles of shaders are illustrated in Figure 2, sketching a toy example where three connected line segments forming a triangle are rendered from three vertices (Figure 2A). A data item with an arbitrary data type is provided for every vertex. In this example, there are two values for the 2D position, and three values for the point's color. The data buffer containing the items for all points is generally stored on the GPU in a *vertex buffer object* (VBO). PyOpenGL can transfer a NumPy array with the appropriate data type to a VBO with minimal overhead.

OpenGL lets us choose the mapping between a data item and variables in the shader program. These variables are called *attributes*. Here, the a_position attribute contains the first two values in the data item, and a_color contains the last three values. The inputs of a vertex shader program consist mainly of attributes, global variables called *uniforms*, and textures. A particularity of a shader program is that there is one execution thread per data item, so that the actual input of a vertex shader concerns a single vertex. This is an example of the Single Instruction, Multiple Data (SIMD) paradigm in parallel computing, where one program is executed simultaneously over multiple cores and multiple bits of data (Almasi and Gottlieb, 1988). This pipeline leverages the massively parallel architecture of GPUs. Besides, GLSL supports conditional branching so that different transformations can be applied to different parts of the data. In Figure 2A, the vertex shader applies the same linear transformation (rotation and scaling) on all vertices.

The vertex shader returns an OpenGL variable called gl_Position that contains the final position of the current vertex in homogeneous space coordinates. The vertex shader can return additional variables called *varying* variables (here, v_color), which are passed to the next programmable stage in the pipeline: the fragment shader.

After the vertex shader, the transformed vertices are passed to the primitive assembly and the rasterizer, where points, lines and triangles are formed out of them. One can choose the mode describing how primitives are assembled. In particular, indexing rendering (not used in this toy example) allows a given vertex to be reused multiple times in different primitives to optimize memory usage. Here, the GL_LINE_LOOP mode is chosen, where lines connecting two consecutive points are rendered, the last vertex being connected to the first.

Finally, once rasterization is done, the scene is described in terms of pixels instead of vector data (Figure 2B). The fragment shader executes on all rendered pixels (pixels of the primitives rather than pixels of the screen). It accepts as inputs varying variables that have been interpolated between the closest vertices around the current pixel. The fragment shader returns the pixel's color.

Together, the vertex shader and the fragment shader offer great flexibility and very high performance in the way data are transformed and rendered on screen. Being implemented in a syntax very close to C, they allow for an unlimited variety of processing algorithms. Their main limitation is the fact that they execute independently. Therefore, implementing interactions between vertices or pixels is difficult without resorting to more powerful frameworks for general-purpose computing on GPUs such as OpenCL (Stone et al., 2010) or CUDA (Nvidia, 2008). These libraries support OpenGL interoperability, meaning that data buffers residing in GPU memory can be shared between OpenGL and OpenCL/CUDA.

### 2.3. Interactive visualization of neurophysiological data

In this section, we apply the techniques described above to visualization of scientific data, and notably neurophysiological signals.

#### 2.3.1. Interactive visualization of 2D data

Although designed primarily for 3D rendering, the OpenGL programmable pipeline can be easily adapted for 2D data processing (using an orthographic projection, for example). Standard plots can be naturally described in terms of OpenGL primitives: scatter points are 2D points, curves consist of multiple line segments, histograms are made of consecutive filled triangles, images are rendered with textures, and so on. However, special care needs to be taken in order to render a large amount of data efficiently.

Firstly, data transfers between main memory and GPU memory are a well-known performance bottleneck, particularly when they occur at every frame (Gregg and Hazelwood, 2011). When it comes to visualization of static datasets, the data points can be loaded into GPU memory at initialization time only. Interactivity (panning and zooming), critical in visualization of big datasets (Shneiderman, 1996), can occur directly on the GPU with no data transfers. Visualization of dynamic (e.g., real-time) datasets is also possible with good performance, as the memory bandwidth of the GPU and the bus is typically sufficient in scientific applications (see also the *Results* section).

The vertex shader is the most adequate place for the implementation of linear transformations such as panning and zooming. Two uniform 2D variables, a scaling factor and a translation factor, are updated according to user actions involving the mouse and the keyboard. This implementation of interactive visualization of 2D datasets is extremely efficient, as it not only leverages the massively parallel architecture of GPUs to compute data transformations, but it also overcomes the main performance bottleneck of this architecture which concerns CPU-GPU data transfers.

The fragment shader is also useful in specific situations where the color of visual objects need to change in response to user input. For instance, the color of points in a scatter plot can be changed dynamically when they are selected by the user. In addition, the fragment shader is essential for antialiased rendering.

#### 2.3.2. Time-dependent neurophysiological signals

The techniques described above allow for fast visualization of time-dependent neurophysiological signals. An intracellular recording, such as one stored in a binary file, can be loaded into system memory very efficiently with NumPy's fromfile function. Then, it is loaded into GPU memory and it stays there as long as the application is running. When the user interacts with the data, the vertex shader translates and scales the vertices accordingly.

A problem may occur when the data becomes too large to reside entirely in GPU memory, which is currently limited to a few gigabytes on high-end models. Other objects residing in the OpenGL context or other applications running simultaneously on the computer may need to allocate memory on the GPU as well. For these reasons, it may be necessary to downsample the data so that only the relevant part of interest is loaded at any time. Such downsampling can be done dynamically during interactive visualization, i.e., the temporal resolution can be adapted according to the current zoom level. There is a trade-off between the amount of data to transfer during downsampling (and thereby the amount of data that resides in GPU memory), and the frequency of these relatively slow transfers.

**Figure 3:**
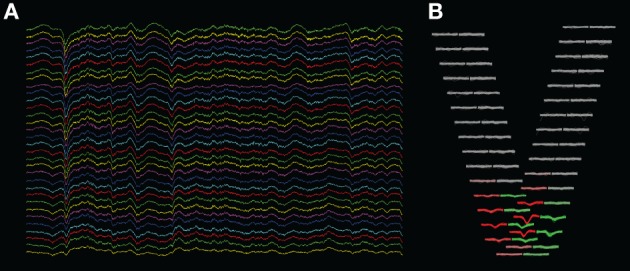
**Screenshots of KwikSkope and KlustaViewa**. **(A)** Raw 32-channel extracellular recordings visualized in KwikSkope. **(B)** In KlustaViewa, spike waveforms, extracted from **(A)**, are displayed in a layout depending on the probe geometry. Channels where waveforms are not detected (amplitude below a threshold), also called *masked channels*, are shown in gray. Two clusters (groups of spikes) are shown here in red and greed.

This technique is implemented in a program which we developed for the visualization of extracellular multielectrode recordings (“KwikSkope,”^6^ Figure 3A). These recordings are sampled at high resolution, can last for several hours, and contain tens to hundreds of channels on high-density silicon probes (Buzsáki, 2004). The maximum number of points to display at once is fixed, and multiscale downsampling is done automatically as a function of the zoom level. Downsampling is achieved by slicing the NumPy array containing the data as follows: data_gpu = data_original[start:end:step,:] where start and end delimit the chunk of data that is currently visible on-screen, and step is the downsampling step. More complex methods, involving interpolation for example, would result in aesthetically more appealing graphics, but in much slower performance as well.

A further difficulty is that the full recordings can be too large to fit in host memory. We implemented a memory mapping technique in KwikSkope based on the HDF5 file format (Folk et al., 1999) and the PyTables library (Alted and Fernández-Alonso, 2003), where data is loaded directly from the hard drive during downsampling. As disk reads are particularly slow [they are much faster on solid-state drives (SDD) than hard disk drives (HDD)], this operation is done in a background thread to avoid blocking the user interface during interactivity. Downsampling is implemented as described above in a polymorphic fashion, as slicing PyTables' Array objects leads to highly efficient HDF5 *hyperslab* selections.^7^

Another difficulty is the fact that support for double-precision floating point numbers is limited in OpenGL. Therefore, a naive implementation may lead to loss of precision at high translation and zoom levels on the x-axis. A classic solution to this well-known problem consists in implementing a floating origin (Thome, 2005). This solution is currently not implemented in Galry nor KwikSkope, but we intend to do so in the future.

#### 2.3.3. Extracellular action potentials

Another example is in the visualization of extracellular action potentials. The process of recovering single-neuron spiking activity from raw multielectrode extracellular recordings is known as “spike sorting” (Lewicki, 1998). Existing algorithmic solutions to this inverse problem are imperfect, so that a manual post-processing stage is often necessary (Harris et al., 2000). The problem is yet harder with new high-density silicon probes containing tens to hundreds of channels (Buzsáki, 2004). We developed a graphical Python software named “KlustaViewa”^8^ for this purpose. The experimenter loads a dataset after it has been processed automatically, and looks at groups of spikes (“clusters”) putatively belonging to individual neurons. The experimenter needs to refine the output of the automatic clustering algorithm. Human decisions include merging or splitting clusters and classifying clusters according to their sorting quality. These decisions are based on the visual shapes of the waveforms of spikes across channels, the automatically-extracted features of these waveforms, and the pairwise cross-correlograms between clusters. The software includes a semi-automatic assistant that guides the experimenter through the process.

We now describe how we implemented the visualization of waveforms across spikes and channels. The waveforms are stored internally as 3D NumPy arrays (Nspikes * Nsamples * Nchannels). As we also know the 2D layout of the probe with the coordinates of every channel, we created a view where the waveforms are organized geometrically according to this layout. In Figure 3B, the waveforms of two clusters (in red and green) are shown across the 32 channels of the probe. This makes it easier for the experimenter to work out the position of the neuronal sources responsible for the recorded spikes intuitively. We needed the experimenter to be able to change the scale of the layout dynamically, as the most visually clear scale depends on the particular dataset and on the selected clusters.

**Figure 4:**
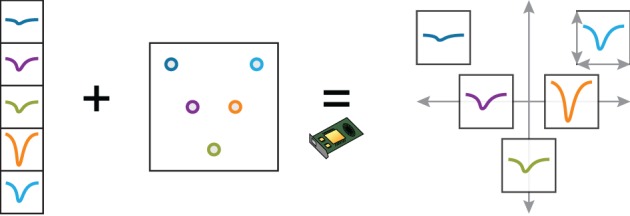
**Dynamic representation of multielectrode extracellular spike wavefoms with the probe geometry**. This example illustrates how a complex arrangement of spike waveforms can be efficiently implemented on the GPU with a vertex shader. The user can smoothly change the local and global scaling of the waveforms. The normalized waveforms, as well as the probe geometry, are loaded into GPU memory at the beginning of the session. The vertex shader applies translation, local and global scaling transformation on the waveform vertices, depending on user-controlled scaling parameters.


When the experimenter selects a cluster, the corresponding waveforms are first normalized on the CPU (linear mapping to [−1, 1]), before being loaded into GPU memory. The geometrical layout of the probe is also loaded as a uniform variable, and a custom vertex shader computes the final position of the waveforms. The scaling of the probe and of the waveforms is determined by four scalar parameters (uniform variables) that are controlled by specific user actions (Figure 4). The GLSL code snippet below (slightly simplified) shows how a point belonging to a waveform is transformed in the vertex shader by taking into account the probe layout, the scaling, and the amount of panning and zooming set by the user.

// Probe layout and waveform scaling.
// wave is a vertex belonging to a
// waveform.
vec2 wave_tr = wave * wave_scale +
channel_pos * probe_scale;
// Interactive panning and zooming.
// gl_Position is the final vertex
// position.
gl_Position = zoom * (wave_tr + pan);


Interactive visualization of these waveforms is fast and fluid, since waveforms are loaded into GPU memory at initialization time only, and the geometrical layout is computed on the GPU.

## 3. Results

We implemented the methods described in this paper in an experimental project called “Galry”^9^ (BSD-licensed, cross-platform, and for Python 2.7 only). This library facilitates the development of OpenGL-based data visualization applications in Python. We focused on performance and designed the library's architecture for our particular needs, which were related to visualization of neurophysiological recordings. The external API and the internal implementation will be improved in the context of a larger-scale project named “Vispy” (see the *Discussion*).

**Figure 5:**
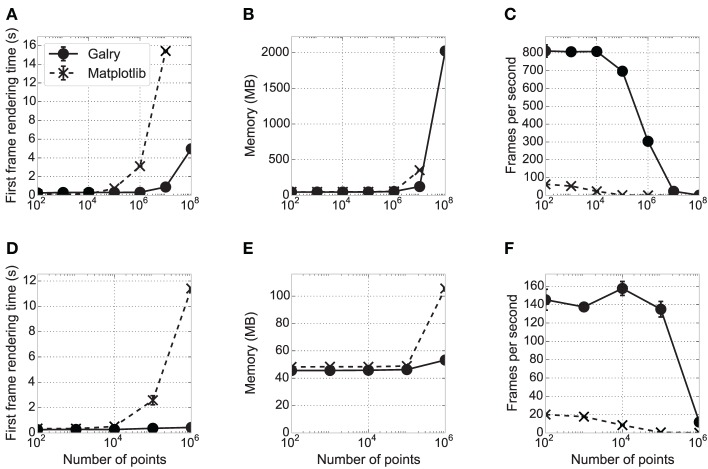
**Performance comparison between matplotlib and Galry.** Ten line plots (white noise time-dependent signals) containing N points in total are rendered in matplotlib 1.3.0 with a Qt4Agg backend (dashed and crosses) and Galry 0.2.0 (solid and discs). All benchmarks are executed three times with different seeds (error bars sometimes imperceptible on the plots). The benchmarks are executed on two different PCs (see details below): PC1 (high-end desktop PC) in **(A–C)**, PC2 (low-end laptop) in **(D–F)**. **(A,D)** First frame rendering time as a function of the total number of points in the plots. **(B,E)** Average memory consumption over time of the Python interpreter rendering the plot. **(C,F)** Median number of frames per second with continuous scaling in the x direction (automatic zooming). Frame updates are requested at 1000 Hz up to a maximum zoom level for a maximum total rendering duration of 10 s. In all panels, some values corresponding to large N could not be obtained because the system ran out of memory. In particular, on PC1, the values corresponding to *N* = 10^8^ could be obtained with Galry but not matplotlib (the system crashed due to RAM usage reaching 100%). PC1: 2012 Dell XPS desktop computer with an Intel Core i7-3770 CPU (8-core) at 3.40 GHz, 8 GB RAM, a Radeon HD 7870 2 GB graphics card, Windows 8 64-bit, and Python 2.7.5 64-bit. PC2: 2012 ASUS VivoBook laptop with an Intel Core i3 CPU (4-core) at 1.4 GHz, 4 GB RAM, an Intel HD 3000 integrated GPU (video memory is shared with system memory), Windows 8.1 64-bit, and Python 2.7.5 64-bit.

In this section, we assess Galry's relative performance against matplotlib using a simple dynamic visualization task (Figure 5, showing the results on a high-end desktop computer, and a low-end laptop). We created identical plots in Galry and matplotlib, which contain ten random time-dependent signals for a total of N points. The code for these benchmarks is freely available online.^10^ The results are saved in a human-readable JSON file and the plots in Figure 5 can be generated automatically.

First, we estimated the first frame rendering time of these plots in Galry and matplotlib, for different values of N. An entirely automatic script creates a plot, displays it, and closes it as soon as the first frame has been rendered. Whereas the first frame rendering times are comparable for medium-sized datasets (10,000–100,000 points), Galry is several times faster than matplotlib with plots containing more than one million points. This is because matplotlib/Agg implement all transformation steps on the CPU (in Python and C). By contrast, Galry directly transfers data from host memory to GPU memory, and delegates rasterization to the GPU through the OpenGL pipeline.

Next, we assessed the memory consumption of Galry and matplotlib on the same examples (using the memory_profiler package^11^). Memory usage is comparable, except with datasets containing more than a few million points where Galry is a few times more memory-efficient than matplotlib. We were not able to render 100 million points with matplotlib without reaching the memory limits of our machine. We did not particularly focus on memory efficiency during the implementation of Galry; an even more efficient implementation would be possible by reducing unnecessary NumPy array copies during data transformation and loading.

Finally, we evaluated the rendering performance of both libraries by automatically zooming in the same plots at a requested frame rate of 1000 frames per second (FPS). This is the aspect where the benefit of GPUs for interactive visualization is the most obvious, as Galry is several orders of magnitude faster than matplotlib, particularly on plots containing more than one million points. Here, the performance of Galry is directly related to the computational power of the GPU (notably the number of cores).

In the benchmark results presented in Figure 5, matplotlib uses the Qt4Agg backend. We also ran the same benchmarks with a non-Agg backend (Wx). We obtained very similar results for the FPS and memory, but the first frame rendering time is equivalent or better than Galry up to *N* = 10^6^ (data not shown).

Whereas we mostly focused on *static* datasets in this paper, our methods as well as our implementation support efficient visualization of *dynamic* datasets. This is useful, for example, when visualizing real-time data during online experiments (e.g., data acquisition systems). With PyOpenGL, transferring a large NumPy array from system RAM to GPU memory is fast (negligible Python overhead in this case). Performance can be measured in terms of memory bandwidth between system and GPU memory. The order of magnitude of this bandwidth is roughly 1 GB/s at least, both by theoretical (e.g., memory bandwidth of a PCI-Express 2.0 bus) and experimental (benchmarks with PyOpenGL, data not shown) considerations. Such bandwidth is generally sufficiently high to allow for real-time visualization of multi-channel digital data sampled at tens of kilohertz.

## 4. Discussion

In this paper, we demonstrated that OpenGL, a widely known standard for hardware-accelerated graphics, can be used for fast interactive visualization of large datasets in scientific applications. We described how high performance can be achieved in Python, by transferring static data on the GPU at initialization time only, and using custom shaders for data transformation and rendering. These techniques minimize the overhead due to Python, the OpenGL API calls, and CPU-GPU data transfers. Finally, we presented applications to visualization of neurophysiological data (notably extracellular multielectrode recordings).

Whereas graphics cards are routinely used for 3D scientific visualization (Lefohn et al., 2003; Rößler et al., 2006; Petrovic et al., 2007), they are much less common in 2D visualization applications (Bailey, 2009). Previous uses of shaders in such applications mainly center around mapping (McCormick et al., 2004; Liu et al., 2013), images or videos (Farrugia et al., 2006). OpenVG^12^ (managed by the Khronos Group) is an open specification for hardware-accelerated 2D vector graphics. There are a few implementations of this API on top of OpenGL.^13^

We compared the performance of our reference implementation (Galry) with matplotlib, the most common visualization library in Python. Even if matplotlib has been optimized for performance over many years, it is unlikely that it can reach the speed of GPU-based solutions. Matplotlib is not the only visualization software in Python; other notable projects include Chaco,^14^ VisTrails (Callahan et al., 2006),^15^ PyQtGraph,^16^ VisVis,^17^ Glumpy,^18^ Mayavi (Ramachandran and Varoquaux, 2011)^19^ (oriented toward 3D visualization). However, none of them is specifically designed to handle extremely large 2D plots as efficiently as possible.

With our techniques, we were able to plot up to 100 million points on a modern computer. One may question the interest of rendering such a large number of points when the resolution of typical LCD screens rarely exceeds a few million pixels. This extreme example was more a benchmark than a real-world example, demonstrating the scalability of the method. Yet, raw datasets with that many points are increasingly common, and, as a first approach, it may be simpler to plot these data without any preprocessing step. Further analysis steps reducing the size and complexity of the graphical objects (subset rendering, downsampling, plotting of statistical quantities, etc.) may be engaged subsequently once the experimenter has gained insight into the nature of the data (Liu et al., 2013). More generally, it could be interesting to implement generic dynamic downsampling methods adapted to common plots.

There are multiple ways our work can be extended. First, we focused on performance (most notably in terms of number of frames per second) rather than graphical quality. We did not implement any OpenGL-based anti-aliasing technique in Galry, as this is a challenging topic (Pharr and Fernando, 2005; Rougier, 2013). Anti-aliased plots result in greater quality and clearer visuals, and are particularly appreciated in publication-ready figures. For example, matplotlib uses anti-aliasing and sub-pixel resolution with the default Agg (Anti-Grain Geometry) backend. High-quality OpenGL-based rendering would be an interesting addition to our methods. Antialiased rendering leads to higher quality but lower performance; end-users could have the choice to disable this feature if they need maximum performance.

Another extension could concern graphical backends. Currently, Galry uses Qt4 as a graphical backend providing an OpenGL context, and it would be relatively easy to support other similar backends like GLUT or wxWidgets. A web-based backend, which would run in a browser, would be highly interesting but challenging. More and more browsers support WebGL, an open specification that lets OpenGL applications written in Javascript run in the browser with hardware acceleration (Marrin, 2011). A web-based backend would enable distributed work, where the Python application would not necessarily run on the same machine as the client. In particular, it would enable visualization applications to run on mobile devices such as smartphones and tablets. Besides, it would increase compatibility, as there are some systems where the default OpenGL configuration is not entirely functional. In particular, some browsers like Chrome and Firefox use the ANGLE library^20^ on Windows to redirect OpenGL API calls to the Microsoft DirectX library, which is generally more stable on Windows systems. Also, it could be possible to “compile” an entire interactive visualization application in a pure HTML/Javascript file, facilitating sharing and diffusion of scientific data. We should note that a web backend would not necessarily require WebGL, as a VNC-like protocol could let the server send continuously locally-rendered bitmap frames to the client.

Another interesting application of a web backend could concern the integration of interactive plots in the IPython notebook. IPython plays a central role in the Python scientific ecosystem (Perez and Granger, 2007), as it offers not only an extended command-line interface for interactive computing in Python, but also a web-based notebook that brings together all inputs and outputs of an interactive session in a single web document. This tool brings reproducibility in interactive computing, an essential requirement in scientific research (Perez et al., 2013). The IPython notebook only supports static plots in version 1.0. However, the upcoming version 2.0 will support Javascript-based interactive widgets, thereby making the implementation of interactive hardware-accelerated plots possible in the notebook.

The aforementioned possible extensions of our work are part of a larger collaborative effort we are involved in, together with the creators of PyQtGraph, VisVis, and Glumpy. This project consists in creating a new OpenGL-based visualization library in Python named “Vispy.”^21^ This future tool (supporting Python 2.6+ and 3.x) will not only offer high-performance interactive visualization of scientific data, thereby superseding our experimental project Galry, but it will also offer APIs at multiple levels of abstraction for an easy and Pythonic access to OpenGL. This library will offer a powerful and flexible framework for creating applications to visualize neuro-anatomical data (notably through hardware-accelerated volume rendering techniques), neural networks as graphs, high-dimensional datasets with arbitrary projections, and other types of visuals. We expect Vispy to become an essential tool for interactive visualization of increasingly large and complex scientific data.

## Funding

This work was supported by EPSRC (EP/K015141) and Wellcome Trust (Investigator award to Kenneth D. Harris).

### Conflict of interest statement

The authors declare that the research was conducted in the absence of any commercial or financial relationships that could be construed as a potential conflict of interest.

## Footnotes

^1^http://matplotlib.org/

^2^http://code.enthought.com/projects/chaco/

^3^http://pyqwt.sourceforge.net/

^4^https://github.com/ContinuumIO/Bokeh

^5^http://www.opengl.org/documentation/implementations/

^6^https://github.com/klusta-team/klustaviewa

^7^http://www.hdfgroup.org/HDF5/doc/UG/12_Dataspaces.html

^8^https://github.com/klusta-team/klustaviewa

^9^https://github.com/rossant/galry

^10^https://github.com/rossant/galry-benchmarks

^11^https://pypi.python.org/pypi/memory_profiler

^12^http://www.khronos.org/openvg/

^13^http://en.wikipedia.org/wiki/OpenVG#On_OpenGL.2C_OpenGL_ES

^14^http://code.enthought.com/chaco/

^15^http://www.vistrails.org/index.php/Mainpage

^16^http://www.pyqtgraph.org/

^17^https://code.google.com/p/visvis/

^18^https://code.google.com/p/glumpy/

^19^http://code.enthought.com/projects/mayavi/

^20^https://code.google.com/p/angleproject/

^21^http://vispy.org/
